# Supplementary figures and images for: Large-sized fossil hamsters from the late Middle Pleistocene Locality 2 of Shanyangzhai, China, and discussion on the validity of Cricetinus and C. varians (Rodentia: Cricetidae)
Source: PeerJ. 2023 Jul 28;11:e15604. doi: 10.7717/peerj.15604 (PMC10389077; doi:10.7717/peerj.15604)

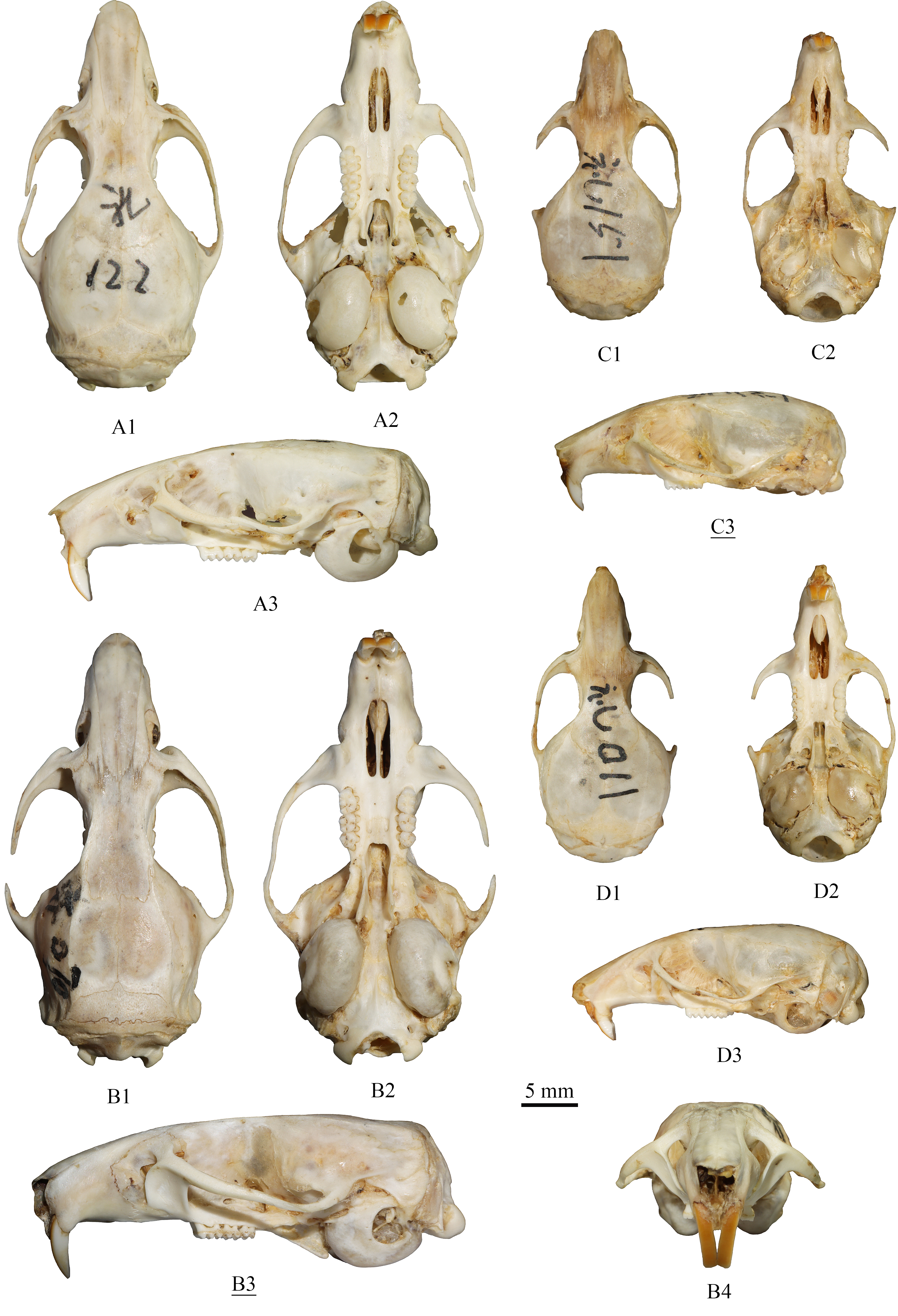

Supplement: Supplemental Information 1 — (A) Tscherskia triton, catalogue number: 礼122; (B) Tscherskia triton, catalogue number: 齐070; (C) Cricetulus barabensis, catalogue number: 礼151; (D) Cricetulus longicaudatus, catalogue number: 礼011. (A1), (B1), (C1), (D1), dorsal view; (A2), (B2), (C2), (D2), ventral view; (A3), (B3), (C3), (D3), lateral view; (B4), anterior view. The underlined label indicates the image has been reversed. [file peerj-11-15604-s001.jpg]

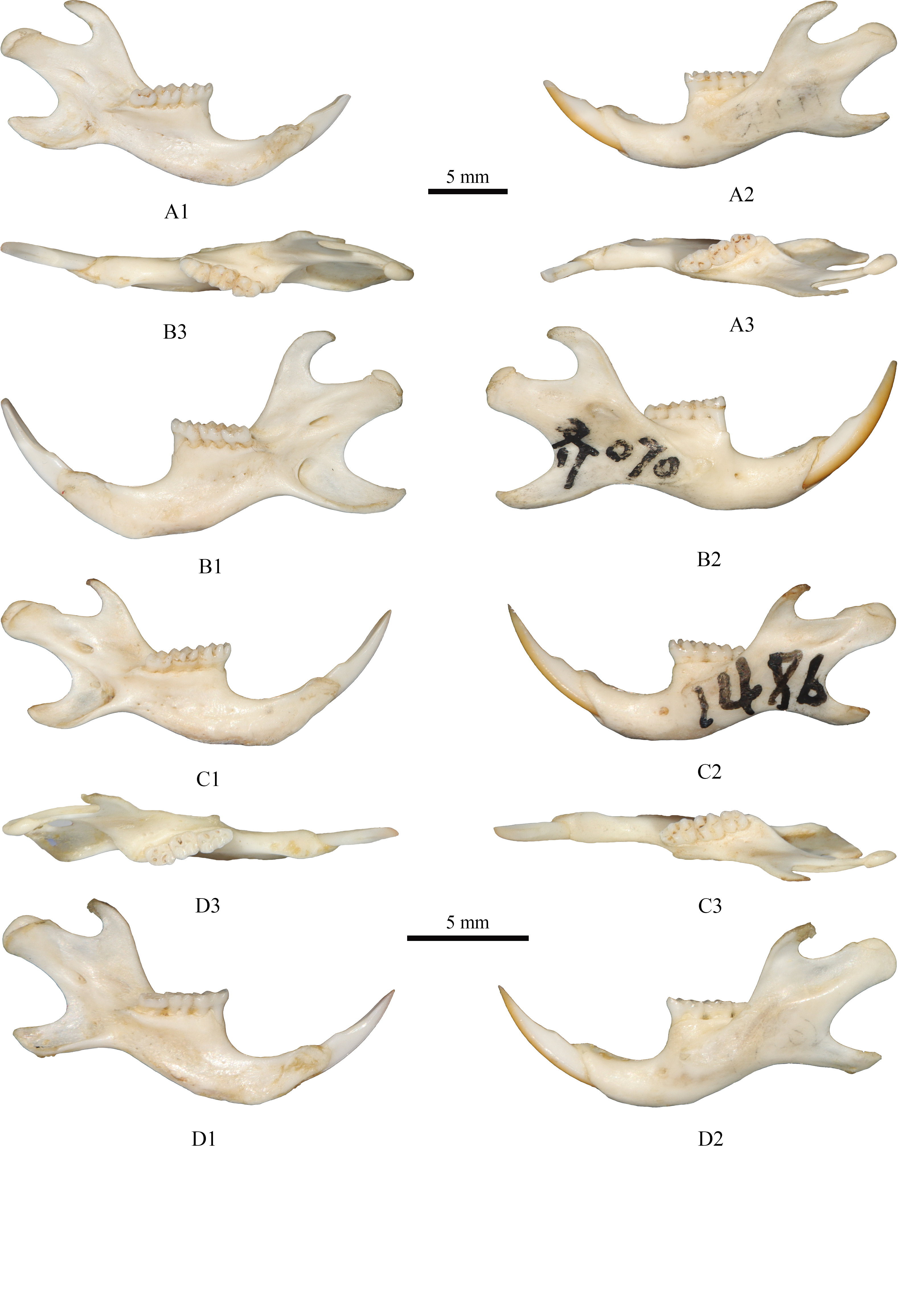

Supplement: Supplemental Information 2 — (A) Tscherskia triton, left mandible, catalogue number: 礼094; (B) Tscherskia triton, right mandible, catalogue number: 齐070; (C) Cricetulus barabensis, left mandible, catalogue number: 148b; (D) Cricetulus longicaudatus, left mandible, catalogue number: 瓦002. (A1), (B1), (C1), (D1), lingual view; (A2), (B2), (C2), (D2), buccal view; (A3), (B3), (C3), (D3), occlusal view. (A) and (B) share one scale bar; (C) and (D) share one scale bar. [file peerj-11-15604-s002.jpg]

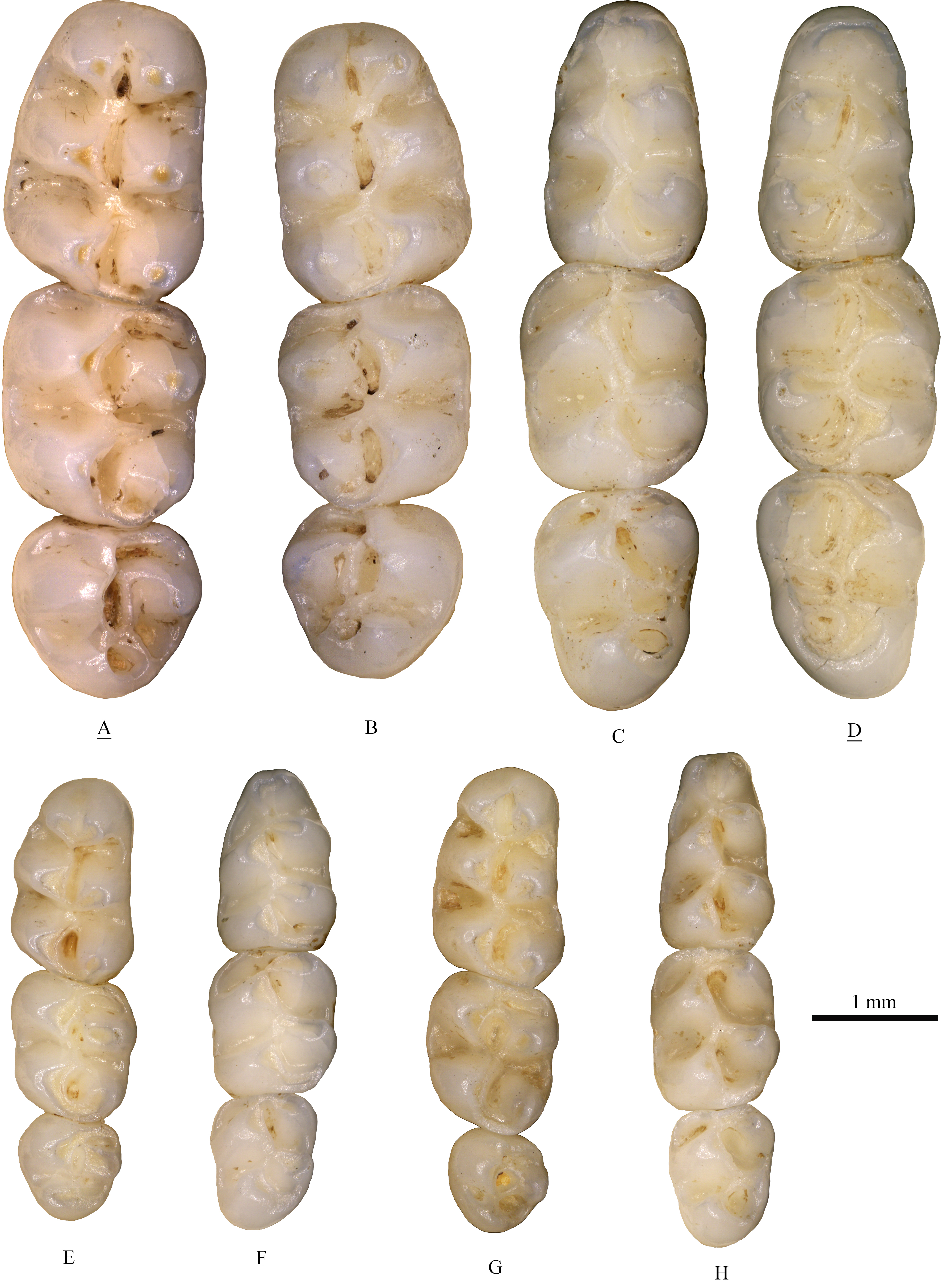

Supplement: Supplemental Information 3 — (A), (B), (C), (D), Tscherskia triton; (E), (F), Cricetulus barabensis; (G), (H), Cricetulus longicaudatus. (A) right upper molars, catalogue number: 158; (B) right upper molars, catalogue number: 127; (C) left lower molars, catalogue number: 齐060; (D) left lower molars, catalogue number: 齐070; (E) left upper molars, catalogue number: 礼144; (F) left lower molars, catalogue number: 礼144; (G) left upper molars, catalogue number: 027; (H) left lower molars, catalogue number: 027. The underlined label indicates the image has been reversed. [file peerj-11-15604-s003.jpg]
